# Mapping Scientific Landscapes and Therapeutic Innovations of Targeted Protein Degradation: A Scientometric Review

**DOI:** 10.3390/pharmaceutics18070887

**Published:** 2026-07-20

**Authors:** Chong Li, Xiangxiu Wang, Anqi He, Tianjie Bao, Weihua Zhuang, Chengqi He, Yonghong Yang

**Affiliations:** 1 Rehabilitation Medicine Center, Institute of Rehabilitation Medicine, West China Hospital, Sichuan University, Chengdu 610041, China; 2 Key Laboratory of Rehabilitation Medicine in Sichuan Province, West China Hospital, Sichuan University, Chengdu 610041, China; 3 Precision Medicine Translational Research Center, West China Hospital, Sichuan University, Chengdu 610041, China

**Keywords:** targeted protein degradation (TPD), PROTAC, molecular glues, CiteSpace, bibliometric

## Abstract

Targeted Protein Degradation (TPD) has emerged as a transformative paradigm in drug discovery, offering a robust strategy to address “undruggable” targets. This study presents the first 25-year longitudinal scientometric analysis (2001–2025) of the TPD field, integrating data from 2750 publications across the Web of Science Core Collection, Scopus, and PubMed to map the global research landscape and therapeutic innovations. The results indicate that TPD research entered an explosive growth phase post-2016. China leads in publication volume (1247 papers), while the USA maintains dominance in citation impact (H-index = 80) and foundational leadership. The Chinese Academy of Sciences and Harvard University were identified as core institutions, with Craig M. Crews confirmed as a pivotal scholar. Thematic analysis reveals a systematic evolution from foundational ubiquitin-proteasome mechanisms to the clinical translation of advanced modalities, including Proteolysis-Targeting Chimeras (PROTACs), molecular glues, and non-ubiquitin-dependent platforms like LYTACs and AUTACs. Clinical viability is evidenced by the FDA approval of the agent ARV-471 for oncology. Despite this progress, critical challenges remain regarding E3 ligase expansion, molecular design optimization, and off-target toxicity. This review provides a data-driven roadmap for future TPD development, bridging the gap between academic output and real-world translational science to guide researchers, clinicians, and industry partners in navigating this dynamic therapeutic frontier.

## 1. Introduction

Over the past twenty-five years, targeted protein degradation (TPD) has redefined therapeutic discovery by enabling the selective and sustained catalytic depletion of pathogenic proteins, rather than merely suppressing their activity [1,2]. Unlike traditional small-molecule inhibitors that rely on continuous target engagement, TPD agents harness endogenous degradation pathways to induce the event-driven knockdown of disease-relevant proteins [3]. This mechanistic distinction confers several advantages, including prolonged pharmacodynamic effects, mitigating traditional occupancy-dependent off-target toxicities, and the capacity to address previously “undruggable” targets such as transcription factors and adaptor proteins [4,5,6].

The TPD field is rapidly expanding, driven by advancements across several key platforms. Representative strategies, notably Proteolysis-Targeting Chimeras (PROTAC), molecular glues, and Lysosome-Targeting Chimeras (LYTACs), have attracted immense interest across academic and industrial settings [7,8,9]. In addition, these approaches have rapidly evolved from conceptual innovations to practical therapeutic modalities, facilitated by progress in E3 ligase identification, ligand optimization, and structure-based molecular design [2,10]. The accelerating number of patents, preclinical studies, and clinical trials has transformed TPD from a niche topic into a central focus of modern chemical biology and drug development [11,12,13].

Despite the rapid advancement of TPD, a systematic and comprehensive overview of the global research landscape and thematic evolution of TPD remains limited. Scientometric and visual analyses offer powerful tools to map scientific knowledge, evaluate research productivity, and uncover emerging frontiers in an interdisciplinary context [14,15,16]. Therefore, applying these approaches to TPD research can reveal the field’s developmental trajectory, highlight influential contributors, and identify evolving therapeutic innovations.

In this study, we conducted a comprehensive scientometric analysis of TPD-related literature published between 2001 and 2025. By integrating quantitative indicators with advanced visualization techniques, we aimed to: (1) examine the global distribution and temporal trends of TPD research; (2) identify leading countries, institutions, and authors; and (3) explore the dynamic evolution of research themes and therapeutic innovations. This work provides an integrated, data-driven perspective on the progression of TPD, offering crucial insights that can guide future scientific exploration and accelerate translational drug development.

## 2. Materials and Methods

### 2.1. Study Design

This study presented a longitudinal bibliometric analysis focused on TPD. The institutional review board of West China Hospital of Sichuan University determined that ethical approval for this study was unnecessary.

### 2.2. Data Source and Retrieval Strategies

Publications from January 2001 to December 2025 were retrieved from the Web of Science Core Collection (WoSCC), Scopus, and PubMed databases. The precise search query, including all terms and combinations, is detailed in Table S1. All publications focused on TPD within this timeframe were included for analysis. To ensure the highest level of accuracy and minimize selection bias, the entire data retrieval and screening process was conducted independently by two experienced researchers. Any discrepancies arising between the two reviewers regarding the inclusion or exclusion of a publication were resolved through discussion and consensus. If a consensus could not be reached, a third senior researcher was consulted to adjudicate the final inclusion decision.

### 2.3. Data Extraction, Standardization, and Analysis

**Figure 1:**
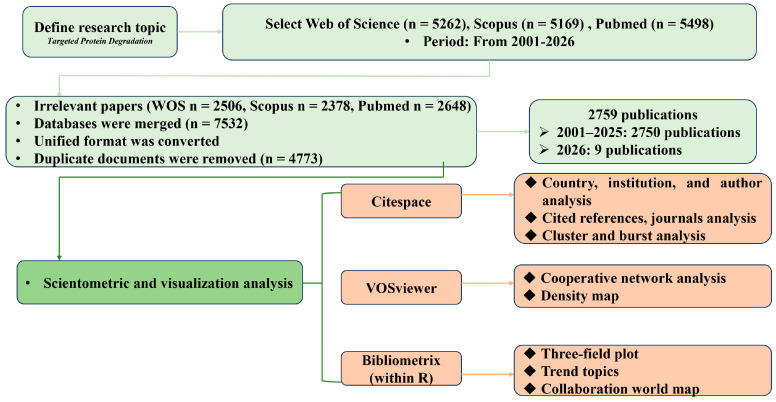
Flow chart of literature data retrieval and processing analysis.

Following the parallel retrieval of heterogeneous metadata from WoSCC, Scopus, and PubMed, a standardized multi-step integration and deduplication protocol was executed. Raw data records were cross-platform homogenized and combined using the “merge DB Sources” function in Bibliometrix (v4.1.2), followed by an algorithmic string-matching filtration and a subsequent refined cleaning via CiteSpace’s (v6.2.R4 Advanced) “Remove Duplicates” utility to systematically eliminate structural redundancies. The finalized, clean dataset was subjected to a chronologically phased evaluation, comprising a 25-year baseline analysis (2001–2025) and a 2026 supplementary frontier mapping. By integrating CiteSpace, VOSviewer (v1.6.21), and Bibliometrix, we dissected the collaborative networks, citation bursts, and macro-thematic evolutions within the field (Figure 1).

## 3. Results and Discussion

### 3.1. General Information and the Trend of Publication Outputs

**Figure 2:**
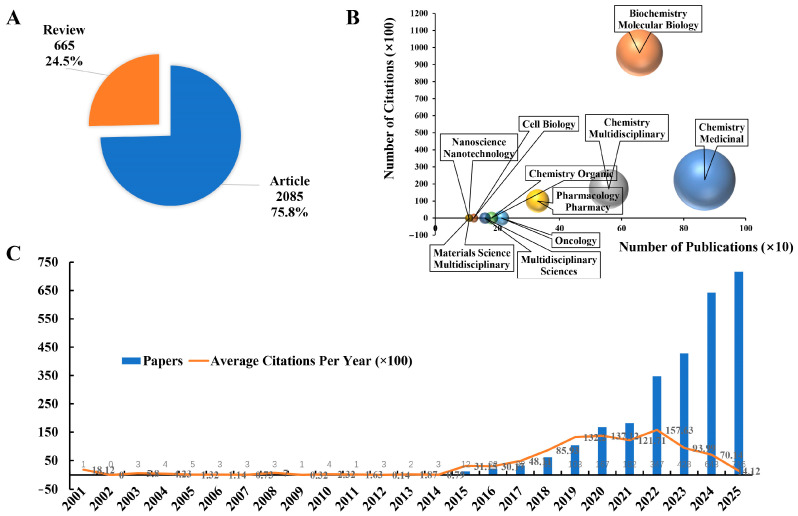
Study characteristics of TPD. (**A**) The pie chart of publication type. (**B**) The bubble chart of publication categories. (**C**) The line chart of annual publications and average citations per year from 2001 to 2025.

A total of 2750 TPD-related papers, consisting of 2085 articles and 665 reviews, were enrolled in this study (Figure 2A). These publications were classified across 75 categories as identified by the WoSCC. Figure 2B presents the top 10 most active categories ranked by publication count. *Medicinal Chemistry* led the output with 868 publications, followed by *Biochemistry Molecular Biology* (n = 658) and *Chemistry Multidisciplinary* (n = 559). In terms of citations, *Biochemistry Molecular Biology* achieved the highest citation count at 96,780, while *Medicinal Chemistry* garnered 22,415 citations. The prominence of *Medicinal Chemistry* in publications and *Biochemistry Molecular Biology* in citations underscores that TPD research is anchored in drug discovery efforts while advancing through molecular-level mechanistic insights. Meanwhile, the representation of interdisciplinary and translational categories like *Oncology and Multidisciplinary Sciences* indicates TPD’s expanding scope in therapeutic applications and cross-field innovations.

The number of TPD-related papers and their average annual citations (scaled by ×100) from 2001 to 2025 are presented in Figure 2C. Prior to 2016, TPD research remained nascent, with the annual publication count consistently below 10 papers and average citations exhibiting low fluctuation. Starting in 2016, the annual number of publications entered a phase of exponential growth, peaking at 716 papers in 2025. This dramatic increase post-2016 clearly signifies a critical inflection point, confirming the growing global academic interest and sustained investment in TPD technology, primarily driven by translational successes in novel drug discovery and refined degradation mechanisms. The peak in average citations around 2021 suggests a period of high academic impact, likely due to breakthroughs in TPD design, efficacy, and clinical potential. The subsequent decline in citations, despite rising publication numbers, may imply that while the field is expanding rapidly, the novelty and impact of individual studies are becoming more dispersed as research diversifies into subfields and applications. Overall, these temporal and categorical trends confirm TPD as a highly dynamic and maturing field in pharmaceutical research, maintaining continuous opportunities for innovation and translational impact.

### 3.2. Timeline of Key Milestones in TPD

**Figure 3:**
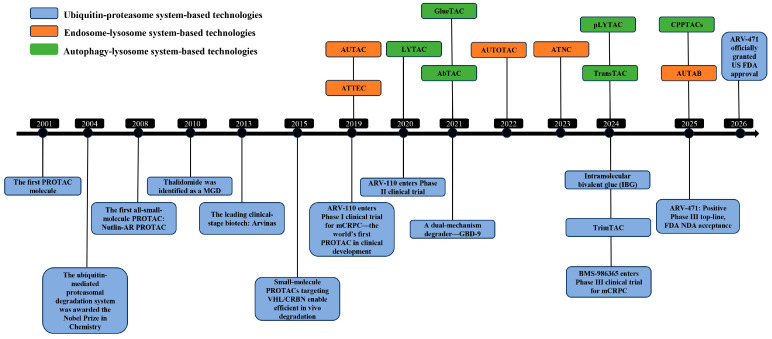
Timeline of key advances in TPD.

The timeline reveals the rapidly accelerating progression of TPD from concept to clinical reality (Figure 3). The foundation was established with the first PROTAC molecule in 2001 [17], utilizing the ubiquitin-proteasome system (UPS), which was validated by the 2004 Nobel Prize for the degradation pathway’s discovery. The subsequent decade marked the translational shift: the development of the first small-molecule PROTAC in 2008 [18] and the identification of Thalidomide as a Molecular Glue Degrader (MGD) in 2010 [19], confirming alternative mechanisms. Clinical viability was cemented by 2015 with the advent of VHL/CRBN-targeting PROTAC [20].

The period from 2019 onward marks explosive clinical and mechanistic expansion. This trajectory culminated in a transformative milestone in May 2026 with the FDA approval of vepdegestrant (Veppanu), the first-ever PROTAC therapy for treating ER+/HER2−, *ESR1*-mutated advanced or metastatic breast cancer. This landmark approval provides definitive clinical validation of the TPD modality, confirming its potential to translate into meaningful survival benefits for patients.

While PROTAC has reached clinical maturity, the TPD landscape has simultaneously undergone a pivotal diversification beyond the UPS. Addressing the inherent limitations of UPS-dependent degradation, the field has witnessed the rapid emergence of non-canonical platforms [21,22,23]. The proposal of endosome-lysosome and autophagy-lysosome pathways has expanded the targetable proteome to extracellular and membrane-bound proteins, as seen in the development of LYTACs and AUTACs [24,25]. Furthermore, the architectural innovation continues with the development of intramolecular bivalent glues (IBG), TrimTACs, and RIPTACs, the latter of which provides a distinct mechanism for degrading targets by sequestering them into non-functional complexes [26,27]. Collectively, this timeline illustrates a two-tier maturation process: the successful clinical validation of UPS-based degraders and the mechanistic emergence of a broader, multi-modal TPD ecosystem. This transition marks the entry of TPD into an era of precision protein modulation, where the clinical success of PROTAC provides a foundation for the next generation of specialized degradation modalities.

### 3.3. Distribution of Countries/Regions

**Figure 4:**
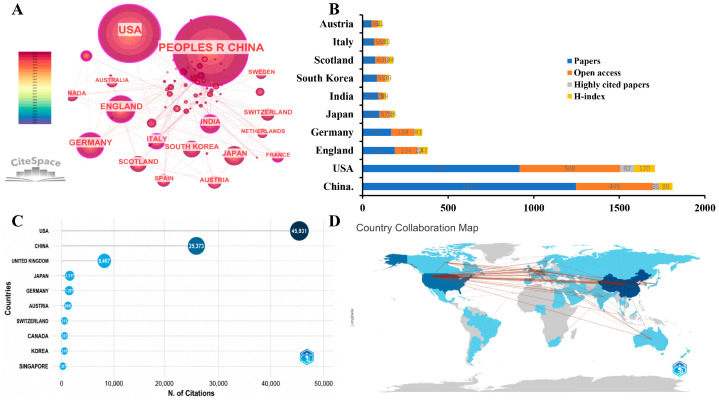
Analysis of countries/regions engaged in TPD research. (**A**) Co-occurrence map. (**B**) Open access papers, highly cited papers, and H-index of top 10 countries/regions in terms of publications; (**C**) Top 10 most cited countries/regions; (**D**) Collaboration world map.

Researchers from 75 countries/regions contributed to the research progress of TPD. In terms of publications, China exhibits overwhelming dominance in publication output (n = 1247, Figure 4A), has a notable H-index (n = 20), and a large number of open-access papers (n = 449), reflecting robust productivity and commitment to knowledge dissemination (Figure 4B). The USA, meanwhile, leads in citations (n = 45,831) and holds the highest H-index (n = 80) with the most highly cited papers (n = 82), indicating its long-standing leadership in impactful TPD research (Figure 4C). In the context of international collaboration, betweenness centrality can serve as a robust indicator to measure the intermediary role of countries in facilitating knowledge exchange and collaborative networks within the TPD research domain [28]. As shown in Figure 4D, the country collaboration map highlights dense ties between the USA (n = 0.56), England (n = 0.37), and China (n = 0.26). These strong global collaborations are essential for resource integration and driving TPD research innovation across borders.

### 3.4. Distribution of Institutions

**Figure 5:**
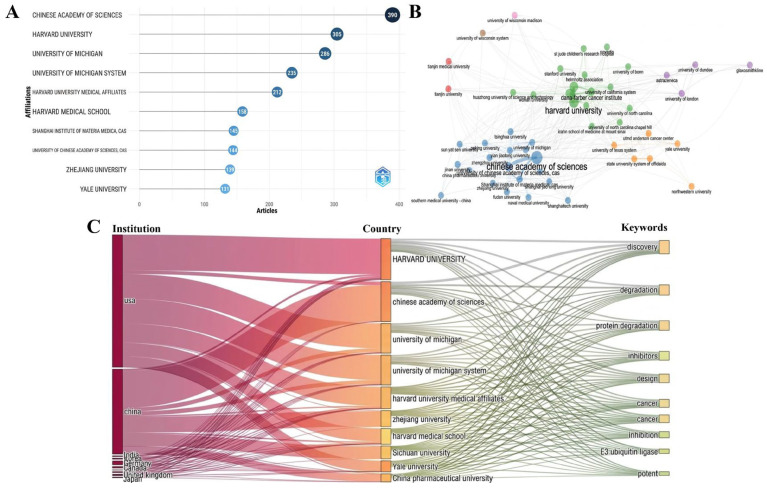
Analysis of institutions engaged in TPD research. (**A**) Top 10 productive institutions. (**B**) Co-collaboration map. (**C**) The three-field plot: middle field: country; left field: institutions; and right field: keywords.

In terms of the distribution of institutions, the *Chinese Academy of Sciences* leads in article count (n = 390), followed by *Harvard University* (n = 305) and the *University of Michigan* (n = 286), demonstrating the former’s prominent productivity and the latter’s sustained academic output in TPD research (Figure 5A). Institutional clusters, as visualized in the co-collaboration map, highlight concentrated strengths of Chinese institutions (*Chinese Academy of Sciences*, *Zhejiang University*) and U.S. institutions (*Harvard University*, *Yale University*), reflecting regionally centered collaborative networks (Figure 5B and Figure S1). The linkage of institutions, countries, and keywords underscores that leading U.S. and Chinese institutions focus on core TPD mechanisms, driving technical advancements (Figure 5C).

The temporal collaboration network visualization reveals the evolving institutional research engagement patterns in TPD from 2001 to 2025 (Figure S2). Pioneering contributions during the early 2000s primarily originated from U.S. institutions such as *Yale University*, the *Howard Hughes Medical Institute*, and the *University of California System*, which were instrumental in establishing the foundational conceptual and methodological framework for TPD technologies. A notable shift in research momentum occurred around 2015, marked by sustained and accelerating activity from institutions globally, particularly the *Chinese Academy of Sciences*, *Harvard University*, and *Zhejiang University*. This chronological transition reflects the field’s progression from nascent academic exploration, largely within the United States academic ecosystem, to a globally collaborative research domain. The subsequent geographical diversification, particularly the intensification of contributions from Chinese research entities over the past 25 years, has significantly expanded the field’s knowledge production capacity.

Additional metrics are summarized in Supplementary Materials, Table S2. *Yale University* demonstrates high innovation (sigma = 20.23), reflecting its early and continued conceptual breakthroughs. The Howard Hughes Medical Institute maintains active collaboration (centrality = 0.19), highlighting its key role as a network hub. St. Jude Children’s Research Hospital exhibits sustained research heat (burst = 7.53), indicating consistent focus on hot topics within the TPD domain. These results confirm the field’s shift from a U.S.-centric origin to a dynamic, globally shared research landscape, where leading institutions contribute specialized expertise to accelerate innovation.

### 3.5. Distribution of Authors

**Figure 6:**
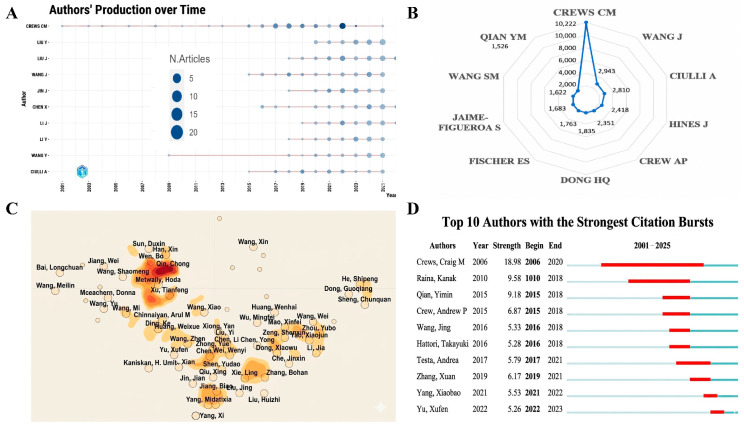
Analysis of authors engaged in TPD research. (**A**) Authors’ production over time. (**B**) Top 10 authors with citations. (**C**) Heatmap. (**D**) Top 10 authors with the strongest citation bursts.

The 2750 included studies were authored by 12,734 researchers. Crews, Craig M demonstrates sustained publication activity since 2001, while Chinese authors such as Liu Y and Liu J, though emerging later, maintain continuous output, indicating the early dominance of U.S. scholars and the subsequent rise in Chinese researchers in TPD research (Figure 6A). In addition, Crews, Craig M leads in citations (n = 10,222), followed by Wang J (n = 2945) and Ciulli A (n = 2810), reflecting his foundational impact and the growing influence of Chinese and European scholars (Figure 6B). Author collaboration clusters, visualized in the heatmap, show core networks formed by U.S. and Chinese scholars, demonstrating international collaboration and regional research concentrations (Figure 6C). Crews, Craig M exhibits the strongest citation burst (n = 18.98, 2006–2020), followed by Raina Kanak and Qian Yimin, highlighting sustained high impact from early contributors and recent emerging scholars (Figure 6D). As shown in Table S3, Crews, Craig M ranks first in H-index (n = 51), total citations (n = 19,596), and number of papers (n = 70), while Chinese authors like Liu Jing and Wang Jing also hold prominent positions, illustrating the dual leadership of U.S. and Chinese scholars in productivity and influence within the TPD field.

### 3.6. Journal Analysis

**Figure 7:**
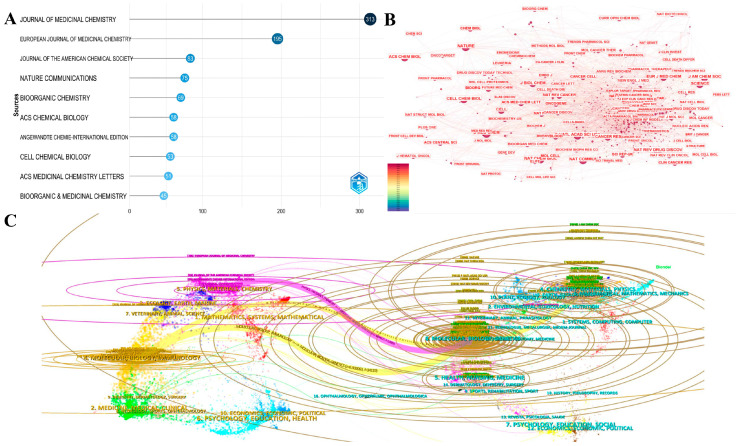
Analysis of journals engaged in TPD research. (**A**) Top 10 productive journals. (**B**) Co-occurrence map of cited journals. (**C**) The dual-map overlay of journals.

The 2750 papers included in this study were published across 505 journals. In terms of document output, *Journal of Medicinal Chemistry* ranks first with 313 papers, followed by the *European Journal of Medicinal Chemistry* with 195 papers. Other highly influential journals, such as *Journal of the American Chemical Society*, have a document output ranging from 30 to 80 (Figure 7A). The top 10 journals primarily involve fields like medicinal chemistry and interdisciplinary chemistry, with Impact Factors ranging from 3.8 to 17 (Table S4), indicating publication in venues of high standing. In terms of citation influence, *J. Med. Chem.* maintains its dominance with a large margin, accumulating 11,605 local citations. High-impact comprehensive journals like *Nature* and *Nature Chemical Biology* also host relatively high citation counts (Figure 7B and Figure S3), reflecting the foundational and breakthrough nature of papers published in these top-tier venues.

The journal overlay map (Figure 7C) visualizes the field’s thematic evolution, highlighting a clear developmental trend: TPD research originated or gained momentum in established fields such as chemical materials and molecular biology before rapidly shifting toward interdisciplinary directions.

Overall, the findings indicate that TPD research is centrally published in specialized medicinal chemistry journals, confirming the field’s focus on small-molecule drug development. Simultaneously, high-impact comprehensive journals actively host interdisciplinary research results, confirming that the field’s focus is undergoing interdisciplinary integration as it matures and expands its mechanistic and therapeutic scope.

### 3.7. Highly Cited Papers and Cited References

**Figure 8:**
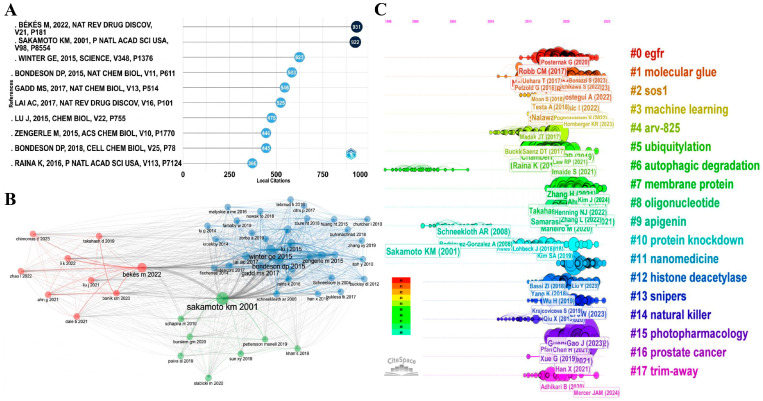
Analysis of highly cited papers and cited references engaged in TPD research. (**A**) Top 10 highly cited papers. (**B**) Co-cited map of cited references. (**C**) Timeline view of cited references.

The 25 highly cited papers in the domain of TPD, predominantly centered on PROTAC technology, encapsulate a trajectory from mechanistic origination to translational breakthroughs (Figure 8A). Pioneering works by Sakamoto et al. [17] established the PROTAC concept by demonstrating targeted protein ubiquitination and degradation. Subsequent structural and chemical biology studies, notably those by Bondeson et al. [20] and Gadd et al. [29], advanced degrader design through structural elucidation and potency optimization, enabling nanomolar-range target degradation and significantly broadening the chemical space of druggable targets. Comprehensive reviews, notably Bekes et al. [1] and Lai et al. [10], synthesized two decades of progress, highlighting PROTAC advantages over traditional small-molecule inhibitors and their burgeoning clinical translation. The high citation metrics of these papers underscore PROTAC’s transformative role in chemical biology and drug discovery—from mechanistic validation to the development of candidates in oncology and beyond—solidifying their status as a paradigm-shifting modality in protein-targeted degradation research (Table 1).

The cited reference network and timeline visualization of TPD research further confirm this evolution (Figure 8B,C). Sakamoto et al. [17] stands out as a crucial foundational node, with subsequent citation clusters expanding across diverse fronts: PROTAC mechanistic advances, molecular glue development, specific targets like EGFR, technological integrations, and novel applications in autophagic degradation and cancer. This thematic landscape clearly reflects TPD’s evolution from conceptual origins to multifaceted explorations in target diversity, technological innovation, and therapeutic translation (Figure S4).

### 3.8. Keyword Analysis

**Figure 9:**
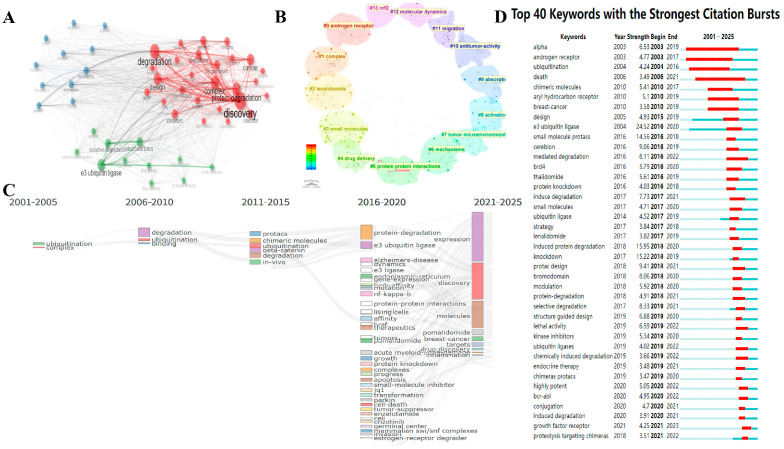
Keywords Analysis engaged in TPD research. (**A**) Co-occurrence map. (**B**) Clusters map. (**C**) Thematic evolution timeline. (**D**) Top 40 keywords with the strongest citation bursts.

The keyword co-occurrence map (Figure 9A) and subsequent cluster analysis (Figure 9B) delineate the primary research themes within the TPD domain. The network visualization highlights the central roles of “degradation,” “ubiquitination,” and “drug discovery,” serving as foundational concepts connecting diverse research fronts. Cluster analysis identifies 14 major themes (Table S5), including core mechanistic clusters like #11 “ubiquitin-ligase” and #4 “molecular glue,” alongside disease-focused clusters such as #0 “androgen receptor” and #10 “anti-tumor activity.” The detailed findings from Cluster #0 (Androgen receptor), for example, confirm the early focus on hormone-dependent cancers using the PROTAC strategy (Table 2, Figure S5). This layered clustering structure reveals the inherent developmental logic of the TPD field: basic ubiquitination and enzymatic mechanisms constitute the disciplinary foundation, while tumor-targeted degradation represents the earliest and most mature translational direction. The clear separation between mechanistic clusters and disease-oriented clusters also reflects an ongoing disciplinary imbalance, wherein fundamental biological research far outpaces diversified therapeutic transformation.

The dynamic thematic evolution map (Figure 9C) reveals a highly structured and accelerating progression in TPD research from 2001 to 2025. Analysis of the first time slice, 2001–2005, confirmed the field’s focus on *Foundational Mechanics*, where themes like “ubiquitination” and the “complex” served as essential *Basic Themes* (Figure S6). Subsequent analysis of 2006–2010 showed a *Diversification of Targets*, as research expanded to include “binding,” “degradation,” and specific therapeutic interests such as “androgen receptor,” initiating the translational trajectory (Figure S7). A significant inflection point was observed in the 2011–2015 slice, where core technological terms—“PROTAC,” “chimeric molecules,” and “in vivo”—rapidly ascended into the *Motor Themes* quadrant, signifying the successful adoption of translational technology (Figure S8). Finally, analysis of the 2016–2025 period confirmed the field’s *Maturity and Therapeutic Expansion*, where foundational terms like “E3 ubiquitin ligase” became highly central *Basic Themes*, while *Motor Themes* broadened dramatically to incorporate applied concepts like “drug discovery,” “optimization,” and broad therapeutic areas (Alzheimer’s, inflammation) (Figures S9 and S10). This systematic, period-by-period evolution confirms TPD’s continuous progression from mechanistic origins to a globally influential, diversified drug discovery platform. Such phased evolution indicates that the maturation of TPD is driven by iterative technological upgrades rather than isolated academic discoveries. The shift from single cancer indications to neurodegenerative and inflammatory diseases demonstrates the field’s maturing translational capability, breaking the original tumor-dominated research paradigm and laying the groundwork for broader clinical application.

The citation burst analysis (Figure 9D) further pinpoints the most active research topics. Early bursts like “ubiquitination” (2004–2016) and “androgen receptor” (2005–2017) confirm their foundational importance. More recent and powerful bursts, such as “induced degradation” (2019–2025), “molecular design” (2020–2025), and “ubiquitin ligase” (2022–2025), indicate a surge of interest in refining degrader efficacy and expanding E3 ligase recruitment. The changing burst hotspots reflect a clear paradigm shift in the TPD community: the field has completed the initial verification of degradation mechanisms and classic target validation and is currently focusing on rational molecular design, ligand optimization, and the exploration of underexploited E3 ligases. This trend also indicates that the limited diversity of recruitable E3 ligases remains the key technical bottleneck restricting large-scale industrial transformation.

Collectively, the thematic landscape confirms the evolution of TPD from basic protein degradation concepts into a multifaceted discipline, driven by both mechanistic refinement and accelerated translational application toward diverse therapeutic targets. Notably, the evolutionary trajectory of the TPD field relies on global collaborative progress, rather than being limited to single-country dominance. Research communities across North America, Europe, Asia, and other regions have made distinct and complementary contributions: pioneering foundational studies on ubiquitin enzymology and structural chemical biology were globally initiated and advanced by multiple academic teams, while the iterative optimization of degrader design, target expansion, and translational transformation benefits from long-term joint efforts of global academic institutions. Furthermore, the booming translational and industrial research of modern TPD is strongly promoted by worldwide pharmaceutical enterprises and biotech companies, which greatly accelerates the clinical translation and industrialization of this emerging technology.

### 3.9. Online Databases and Modelling Tools for TPD

The accelerating complexity of rational TPD degrader design and the rapid expansion of experimental data have driven the continuous development of specialized online databases and computational modeling tools, forming a gradually mature computational auxiliary ecosystem for TPD research (Table 3). Representative open-access databases, including TPDdb and PROTAC-DB, systematically integrate experimentally verified degrader structures, binding affinity data, and target information, providing fundamental data support for mechanism analysis and compound screening. Meanwhile, emerging computational modeling tools such as PROTACable and PROSeRetaC enable the structural prediction of ternary complexes and automated de novo molecular design, effectively compensating for the high cost and low throughput of traditional experimental screening.

Nevertheless, these available resources exhibit distinct differences in technical maturity, data coverage, and practical applicability, rather than providing uniform and comprehensive support. Current TPD-related databases vary greatly in data update frequency and data quality: early established platforms possess large-scale accumulated datasets but suffer from delayed updates and insufficient inclusion of newly reported degraders, while newly developed databases feature timely data iteration but lack systematic integration of historical experimental evidence. In terms of modeling tools, most existing algorithms focus on ternary complex docking and structural optimization, yet they still face inherent limitations in predicting cell permeability, pharmacokinetic properties, and off-target effects, which restricts their practical application in preclinical translational research.

From a global developmental perspective, these data resources and computational platforms are jointly constructed and iteratively optimized by academic institutions worldwide, reflecting the global collaborative progress of TPD computational research. At present, computational tools are widely adopted in early-stage academic screening, while industrial pharmaceutical research tends to rely on self-built proprietary databases and optimized commercial algorithms to improve the success rate of degrader development. This divergence further indicates that the current public TPD tool system still has gaps in industrial adaptability and translational robustness. Overall, although existing databases and modeling tools have greatly improved the efficiency of early TPD molecular design, further algorithm optimization, data standardization, and multi-dimensional performance prediction capability upgrades are still required to meet the growing demands of precision degrader development and clinical transformation.

### 3.10. Clinical Landscape and Modality Diversification

The clinical pipeline of TPD therapeutics is advancing at an accelerated pace, transitioning from exploratory clinical translation toward a productive harvest era, as summarized in Table 4. A landmark regulatory breakthrough occurred on 1 May 2026, with the US FDA’s approval of vepdegestrant (VEPPANU, ARV-471), an oral ER-targeting PROTAC co-developed by Arvinas and Pfizer. Approval was underpinned by pivotal Phase III VERITAC-2 trial (NCT05654623) outcomes, in which vepdegestrant reduced the risk of disease progression or all-cause mortality by 43% versus fulvestrant standard-of-care for patients with *ESR1*-mutated, ER+/HER2− advanced breast cancer. This approval marks the world’s first regulatory clearance for heterobifunctional protein degraders and clinically validates the translational and commercial feasibility of the TPD therapeutic modality.

Beyond breast oncology, PROTAC-based candidates predominate late-stage clinical development for other hormone-dependent malignancies. For metastatic castration-resistant prostate cancer (mCRPC), two second-generation androgen receptor (AR) degraders, luxdegalutamide (ARV-766, partnered with Novartis) and bavdegalutamide (ARV-110), have exhibited robust clinical efficacy by overcoming common ligand-binding domain mutations that confer therapeutic resistance to conventional small-molecule AR antagonists. In parallel, next-generation MGDs are approaching key regulatory endpoints for hematological malignancies; lead candidates, including BMS-986365, exert potent target engagement and elicit durable clinical responses, prolonging progression-free survival (PFS) as single agents or combination regimens in heavily pretreated, refractory patient populations.

Notably, the clinical landscape of TPD is diversifying continuously in terms of mechanistic modalities and therapeutic indications. In addition to canonical UPS-dependent degraders recruiting CRBN/VHL E3 ubiquitin ligases, lysosome- and autophagy-dependent chimeric molecules exemplified by ATGi-101 have entered Phase I clinical testing for advanced solid tumors. Furthermore, TPD development is expanding beyond oncology into non-malignant disorders. Early 2026 clinical data from degrader candidates against disease-driving intracellular kinases and pathological protein aggregates have achieved dose-dependent reduction in target proteins in human cerebrospinal fluid (CSF), laying a solid foundation for neurodegenerative disease treatment. Concurrent ongoing clinical trials in androgenetic alopecia and atopic dermatitis further demonstrate that TPD has evolved from laboratory chemical tools into a transformative therapeutic platform applicable across multiple disease fields.

### 3.11. Emerging Frontiers and Future Challenges

Recent publications highlight the dynamic and expansive nature of TPD research, focusing on overcoming core challenges and broadening applicability. Key advancements include the design of highly potent PROTAC targeting difficult receptors like EGFR triple mutants and ER for refractory breast cancer, demonstrating success against drug resistance mechanisms [162,163]. Furthermore, significant innovation is seen in modality diversification for enhanced CAR-T therapy [164], and the repurposing of TPD for non-oncology applications, such as antiviral strategies [165] and targeting immune checkpoints like LAG-3 [166]. Mechanistic insights continue to advance, with studies exploring mini-PROTACs to improve drug-like properties [167] and analyzing the paradoxical toxicity of neo-substrate degradation in CRBN-based systems [168]. These studies underscore the field’s rapid pivot toward clinical optimization and novel mechanism exploitation.

**Figure 10:**
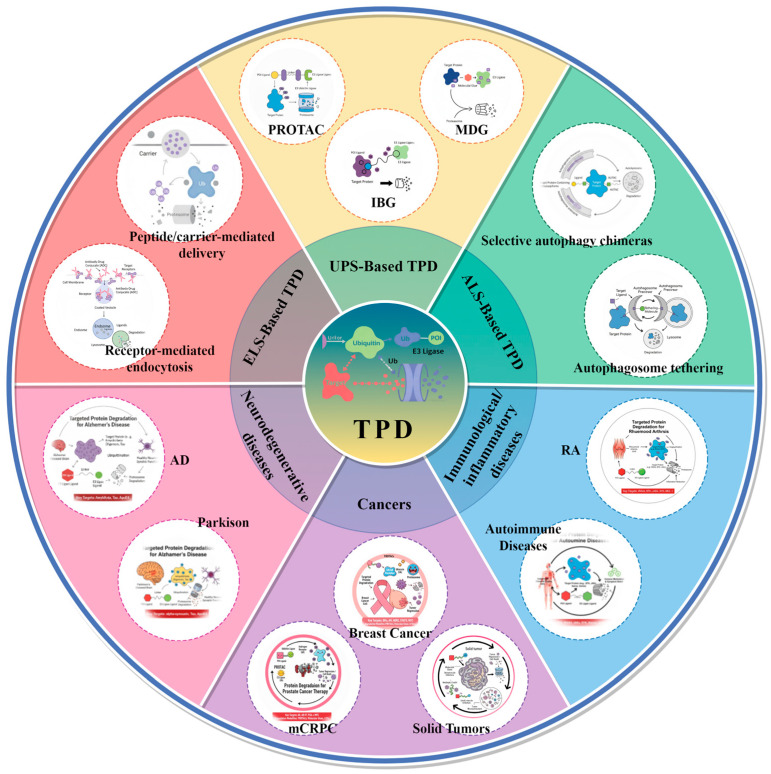
Schematic overview of TPD and its applications across disease areas and technical strategies. Created by Figdraw (ID: WTSYYabb6a).

Despite rapid progress, TPD faces critical challenges across the entire development spectrum. In drug discovery, key obstacles involve expanding the E3 ligase repertoire beyond CRBN and VHL, and improving the physicochemical properties of large PROTAC molecules to enhance cell permeability and oral bioavailability. Crucially, while the catalytic nature of TPD mitigates classic occupancy-driven kinome cross-reactivity, mitigating the unique risk of neo-substrate degradation remains a central unsolved paradox. For disease applications, the primary challenge is to successfully broaden the focus beyond oncology to non-cancer fields like antiviral therapy and neurodegeneration, which often require crossing the blood–brain barrier. Finally, in Clinical Translation, challenges center on validating novel non-UPS modalities (LYTACs, AUTACs) and optimizing targeted delivery strategies to ensure systemic efficacy and minimize potential resistance mechanisms (Figure 10).

## 4. Conclusions

This extensive bibliometric analysis provides a comprehensive scientometric overview of the TPD field, confirming its systematic evolution and rapid acceleration over 25 years. Research output is led by the USA (highest impact) and China (highest volume), with Yale University and Craig M. Crews established as foundational pioneers. Thematic analysis confirms a transition from early focus on ubiquitination mechanics to the translational dominance of PROTAC and MGD, with a current emphasis on drug design and optimization. TPD has successfully entered the clinical landscape, evidenced by the dominance of PROTAC in Phase III trials for cancers and the crucial expansion into non-oncology applications. To fulfill its therapeutic promise, the field should concentrate on expanding the E3 ligase repertoire, addressing physicochemical property challenges, mitigating neo-substrate toxicity, and accelerating the validation of novel non-UPS degradation platforms in clinical settings.

## Figures and Tables

**Table 1:** The detailed information on the 25 highly cited papers.

Publication	Main Find	Corresponding Author	Times Cited	Journal
BÉKÉS M et al. (2022) [1]	This Review summarizes two decades of PROTAC progress and outlines key future directions.	Crews, Craig M	1952	Nature Reviews Drug Discovery
Sakamoto, KM et al. (2001) [17]	Protac-1 mediates MetAP-2 ubiquitination/degradation via SCFβ-TRCP, with potential therapeutic applications.	Sakamoto, KM	1797	PNAS USA
Lai, AC et al. (2017) [10]	PROTAC-mediated protein degradation offers key advantages over traditional small molecules and draws pharmaceutical industry interest.	Crews, Craig M	1083	Nature Reviews Drug Discovery
Bondeson, DP et al. (2015) [20]	Improved PROTACs catalytically degrade target proteins at nanomolar concentrations with broad tissue distribution and potent knockdown, combining small-molecule advantages with RNAi/CRISPR-like efficacy.	Bondeson, Daniel P	971	Nature Chemical Biology
Lu, J et al. (2015) [30]	ARV-825, a cereblon-recruiting PROTAC, degrades BRD4 more effectively than small-molecule inhibitors, leading to superior c-MYC suppression, antiproliferative activity, and apoptosis induction in Burkitt’s lymphoma.	Crews, Craig M	895	Chemistry and Biology
Gadd, MS et al. (2017) [29]	Crystal structure of MZ1-VHL-Brd4(BD2) complex explains PROTAC-induced selective Brd4 degradation, enabling AT1 design.	Ciulli, Alessio	854	Nature Chemical Biology
Zengerle, M et al. (2015) [31]	PROTAC MZ1 selectively degrades BRD4 over BRD2/3, inducing distinct transcriptional responses and enabling selective BRD4 targeting studies.	Ciulli, Alessio	794	ACS Chemical Biology
Banik, SM et al. (2020) [32]	LYTACs enable targeted degradation of extracellular and membrane-associated proteins, with broad research and therapeutic implications.	Bertozzi, Carolyn R	761	Nature
Burslem, GM et al. (2020) [4]	Reviewed the PROTAC technology, its drug discovery applications, development workflow, pros/cons, and comparisons with RNAi/genome editing.	Crews, Craig M	735	Cell
Schapira, M et al. (2019) [2]	Reviewed lessons from TPD.	Schapira, Matthieu	652	Nature Reviews Drug Discovery
Raina, K et al. (2016) [33]	The first to demonstrate efficacy with a small-molecule BET degrader in a solid-tumor malignancy.	Coleman, Kevin G	646	PNAS USA
Bondeson, DP et al. (2018) [34]	Highlights design guidelines for generating potent PROTACs.	Crews, Craig M	642	Cell Chemical Biology
Zhao, L et al. (2022) [9]	Summarized recent advances of major TPD technologies.	Jia, Da	521	Signal Transduction and Targeted Therapy
Toure, M et al. (2016) [35]	Reviewed strategies of small-molecule PROTACS.	Crews, Craig M	519	Angewandte Chemie-Iternational Edition
Burslem, GM et al. (2018) [36]	An RTK of TPD Case Study	Crews, Craig M	493	Cell Chemical Biology
Lai, AC et al. (2016) [37]	As a starting point for PROTAC development, both the target ligand and the recruited E3 ligase should be varied to rapidly generate a PROTAC with the desired degradation profile.	Crews, Craig M	492	Angewandte Chemie-Iternational Edition
Khan, S et al. (2019) [38]	DT2216 may be developed as a safe first-in-class anticancer agent targeting BCL-X-L.	Zhou, Daohong	473	Nature Medicine
Li, K et al. (2022) [6]	Highlighted important milestones and briefly discussed lessons learned about TPD.	Crews, Craig M	470	Chemical Society Reviews
Sun, XY et al. (2019) [39]	More efforts are needed to gain to achieve deeper insight into the efficacy and safety of PROTACs in the clinic.	Rao, Yu	470	Signal Transduction and Targeted Therapy
Schneekloth, AR et al. (2008) [18]	Developed a heterobifunctional all-small molecule PROTAC capable of inducing proteasomal degradation of the androgen receptor.	Crews, Craig M	463	Bioorganic and Medicinal Chemistry Letters.
Dale, B et al. (2021) [40]	Offer a comprehensive account of the most promising degraders in development as cancer therapies to date.	Jin, Jian	448	Nature Reviews Cancer
Petzold, G et al. (2016) [41]	Provided a mechanistic explanation for the selective efficacy of lenalidomide in del(5q) MDS therapy.	Thomae, Nicolas H	440	Nature
Nowak, RP et al. (2018) [42]	Provided a conceptual framework for the development of heterobifunctional ligands	Fischer, Eric S	438	Nature Chemical Biology
Neklesa, TK et al. (2017) [43]	PROTACs, bifunctional small molecules enabling targeted intracellular protein degradation, show promising preclinical data but face delivery/bioavailability challenges for clinical translation.	Neklesa, Taavi K.	437	Pharmacology and Therapeutics
Takahashi, D et al. (2017) [44]	UTAC provides a new modality for research on autophagy-based drugs.	Arimoto, Hirokazu	435	Molecular Cell

**Table 2:** Detailed information on the top 3 publications from each of the 14 clusters.

Cluster Label	Publication	Main Find	Corresponding Author	Times Cited	Journal
Androgen receptor	Chirnomas, D et al. (2023) [11]	Estrogen receptor-targeting heterobifunctional degraders show promise in hormone-dependent breast cancer by degrading this oncogenic protein via the ubiquitin-proteasome system.	Crews, Craig M	385	Nature Reviews Clinical Oncology
	Sakamoto, KM et al. (2003) [45]	An estradiol-based PROTAC mediates ubiquitination and degradation of ERα in vitro.	Deshaies, RJ	332	Molecular and Cellular Proteomics
	Rodriguez-gonzalez, A et al. (2008) [46]	Estradiol-conjugated PROTAC-B degrades ERα via proteasome to inhibit ERα-dependent breast cancer cell proliferation.	Sakamoto, K M	170	Oncogene
Complex	BÉKÉS M et al. (2022) [1]	This Review summarizes two decades of PROTAC progress and outlines key future directions.	Crews, Craig M	1952	Nature Reviews Drug Discovery
	Gadd, MS et al. (2017) [29]	Crystal structure of MZ1-VHL-Brd4(BD2) complex explains PROTAC-induced selective Brd4 degradation, enabling AT1 design.	Ciulli, Alessio	854	Nature Chemical Biology
	Bondeson, DP et al. (2018) [34]	Highlights design guidelines for generating potent PROTACs.	Crews, Craig M	642	Cell Chemical Biology
Lenalidomide	Nowak, RP et al. (2018) [42]	Lenalidomide mediates plastic CRBN-BRD4 interactions to support rational design of selective BET-targeting degraders.	Fischer, Eric S	438	Nature Chemical Biology
	Chamberlain, PP et al. (2019) [47]	Lenalidomide validates targeted protein degradation and supports PROTAC development.	Hamann, Lawrence G	356	Nature Chemical Biology
	Zhang, X et al. (2019) [48]	Electrophilic PROTACs target DCAF16 to enable nuclear protein degradation with minimal E3 ligase perturbation.	Cravatt, Benjamin F	330	Nature Chemical Biology
Small molecules	Chirnomas, D et al. (2023) [11]	Small molecules selectively eliminate disease-related proteins via the ubiquitin-proteasome system, emerging as promising cancer therapeutics in clinical trials.	Crews, Craig M	385	Nature Reviews Clinical Oncology
	Nalawansha, DA et al. (2020) [49]	Small molecules enable targeted protein degradation via the ubiquitin-proteasome system, advancing precision medicine.	Crews, Craig M	333	Cell Chemical Biology
	Huang, H et al. (2018) [50]	Small molecules recruit E3 ligases to selectively degrade kinases, enabling target triage for drug development.	Gray, Nathanael S	310	Cell Chemical Biology
Drug delivery	He, S et al. (2021) [51]	Aptamer-PROTAC conjugates enhance tumor targeting, BET degradation, and antitumor efficacy while reducing toxicity.	Sheng, Chunquan	225	Angewandte Chemie-Iternational Edition
	Liu, J et al. (2021) [52]	Folate-conjugated PROTACs enable folate receptor-dependent selective protein degradation in cancer cells.	Wei, Wenyi	203	Journal of the American Chemical Society
	He, M et al. (2022) [4]	Targeted drug delivery strategies (e.g., aptamer/folate conjugation) enhance PROTAC tumor selectivity and reduce off-tissue toxicity.	Rao, Yu	185	Signal Transduction and Targeted Therapy
Protein–protein interactions	Diehl, CJ et al. (2022) [53]	Small-molecule VHL ligands enable PROTAC-mediated protein–protein interactions between VHL E3 ligase and neo-substrates for targeted degradation.	Ciulli, Alessio	127	Chemical Society Reviews
	Soares, P et al. (2018) [54]	Structure-guided VHL inhibitors disrupt VHL:HIF-α protein–protein interactions and serve as ligands for PROTACs.	Ciulli, Alessio	119	Journal of Medicinal Chemistry
	Domostegui, A et al. (2022) [55]	Molecular glue degraders induce protein–protein interactions between target proteins and E3 ligases for targeted degradation.	Mayor-ruiz, Cristina	119	Chemical Society Reviews
Mechanisms	Chirnomas, D et al. (2022) [11]	Heterobifunctional degraders harness the ubiquitin-proteasome system to eliminate disease-related proteins via an event-driven mechanism.	Crews, Craig M	385	Nature Reviews Clinical Oncology
	Han, X et al. (2019) [56]	ARD-69, a potent PROTAC, induces dose- and time-dependent AR degradation to suppress prostate cancer cell growth.	Wang, Shaomeng	309	Journal of Medicinal Chemistry
	Liu, J et al. (2021) [57]	TF-PROTACs link TF-specific DNA oligonucleotides to E3 ligase ligands to induce selective TF degradation via the UPS.	Wei, Wenyi	177	Journal of the American Chemical Society
Tumor microenvironment	Zhang, C et al. (2022) [58]	Nano-PROTACs mediate CatB-activated COX-1/2 degradation and phototherapy to reprogram the immunosuppressive tumor microenvironment.	Pu, Kanyi	108	Angewandte Chemie-Iternational Edition
	Zhang, H et al. (2022) [59]	pH/GSH-responsive nano-PROTAC CREATE eliminates lung cancer cells and TAMs to remodel the tumor microenvironment.	Zhang, Lingmin	79	Advanced Science
	Zhang, S et al. (2024) [60]	RPB7H nanosensitizer (PROTAC prodrug + HfO_2_) targets BRD4-RAD51AP1 axis to enhance RT-induced DNA damage and remodel HNSCC tumor microenvironment.	Xu, Zhiai	46	Advanced Materials
Activation	Buckley, DL et al. (2015) [61]	Small-molecule VHL-based HaloPROTACs activate targeted degradation of HaloTag7 fusion proteins.	Crews, Craig M	345	ACS Chemical Biology
	Bond, MJ et al. (2020) [62]	LC-2 (KRAS(G12C)-targeting PROTAC) activates VHL-mediated degradation of endogenous KRAS(G12C) to suppress MAPK signaling.	Crews, Craig M	309	ACS Central Science
	Buhimschi, AD et al. (2018) [63]	MT-802 (BTK PROTAC) activates cereblon-mediated degradation of wild-type/C481S BTK.	Crews, Craig M	305	Biochemistry
Absorption	Price, E et al. (2024) [64]	ETR (EPSA/TPSA ratio) and AB-MPS enable absorption prediction for bRo5 compounds including PROTACs.	Degoey, David	40	Journal of Medicinal Chemistry
	Apprato, G et al. (2024) [65]	2D/3D descriptors and hybrid strategies guide discovery of orally bioavailable PROTACs.	Kihlberg, Jan	40	Drug Discovery Today
	Schade, M et al. (2024) [66]	eHBD ≤ 2 (Rule-of-oral-PROTACs) and solution conformations govern PROTAC oral absorption.	Wilson, David	27	Journal of Medicinal Chemistry
Antitumor activity	Dong, G et al. (2021) [67]	Molecular glues induce proximity-dependent protein degradation with distinct antitumor activities.	Sheng, Chunquan	235	Journal of Medicinal Chemistry
	Robb, CM et al. (2017) [68]	CRBN-recruiting CDK9 PROTAC mediates selective CDK9 degradation with antitumor potential.	Rana, Sandeep	183	Chemical Communications
	Bondeson, DP et al. (2017) [69]	Small-molecule-mediated selective protein degradation offers antitumor potential by targeting previously undruggable proteins.	Crews, Craig M	149	Annual Review of Pharmacology and Toxicology
Migration	Li, Q et al. (2023) [70]	The constituents and applications of different nonconventional PROTACs are discussed.	Canhua Huang	13	European Journal of Medicinal Chemistry
	Teufelsbauer, M et al. (2024) [71]	BET-targeting PROTACs (ARV-771/MZ1) and inhibitor JQ1 suppress migration of TNBC cells.	Hamilton, Gerhard	4	Breast Cancer Research and Treatment
	Xu, M et al. (2024) [72]	FAK-targeted PROTAC F2 inhibits cancer cell migration via AKT/ERK pathway suppression and EMT modulation.	Zhang, Xiong-wen	4	Acta Pharmacologica Sinica
Molecular dynamics	Poongavanam, V et al. (2022) [73]	Molecular dynamics simulations predict cereblon PROTAC cell permeability by evaluating folded conformations and solvent-accessible polar surface area.	Kihlberg, Jan	78	Journal of Medicinal Chemistry
	Jimenez, DG et al. (2022) [74]	Steered molecular dynamics-derived conformer descriptors fail to model PROTAC thermodynamic solubility.	Caron, Giulia	64	Journal of Medicinal Chemistry
	Kumar, H et al. (2024) [75]	Molecular dynamics/steered molecular dynamics simulations characterize ternary complex dynamics/stability to guide PROTAC design.	Sobhia, Masilamani Elizabeth	9	ACS Medicinal Chemistry Letters
NRF2	Park, SY et al. (2023) [76]	KEAP1-targeting PROTAC SD2267 induces CRBN-mediated KEAP1 degradation to activate NRF2 and attenuate oxidative stress.	Oh, Seung Hyun	21	Redox Biology
	Chen, H et al. (2023) [77]	PROTAC 14 mediates CRBN-dependent KEAP1 degradation to activate NRF2-driven antioxidant responses.	Sleebs, Brad E	21	Redox Biology
	Ji, J et al. (2023) [78]	CRBN-recruiting PROTAC C2 mediates selective degradation of Nrf2-MafG heterodimer to inhibit Nrf2-ARE signaling.	Jiang, Zhengyu	21	Journal of Medicinal Chemistry

**Table 3:** Online Databases and Modelling tools for TPD Research and Development.

Database/Models	Institution	Core Function	Accessibility/URL	Publication
**Database**				
TPDdb	Xian Jiaotong Liverpool Univ	The comprehensive database of targeted protein degraders	https://idrblab.org/TPDdb/ (accessed on 14 October 2025)	Qin XR et al. (2025) [79]
PROTAC-PatentDB	University of Macau	A PROTAC Patent Compound Dataset	http://protacpatentdb.com (accessed on 19 November 2025)	Cai H et al. (2025) [80]
PROTAC-DB	Zhejiang University	A web-based open-access database integrates structural information and experimental data of PROTACs.	http://cadd.zju.edu.cn/protacdb/ (accessed on 8 January 2021)	Weng, GQ et al. (2024) [81,82,83]
PROTAC-Databank	ShanghaiTech University	Optimized tools for PROTAC design, streamlining the screening process and reducing both time and costs.	https://bailab.siais.shanghaitech.edu.cn/services/deepprotac-db (accessed on 15 September 2024)	Bai F et al. (2024) [84]
ELIOT	University of Perugia	A platform to navigate the E3 pocketome and aid the design of new PROTACs	https://eliot.moldiscovery.com (accessed on 20 July 2022)	Palomba M et al. (2023) [85]
PROTACpedia	NA	1190 PROTAC entries	https://protacdb.weizmann.ac.il/ptcb/main (accessed on 25 September 2020)	NA
MeDBA	Sichuan University	1415 PROTAC entries targeting metalloenzymes, including PROTAC structure, DC50, E3 ligase, etc.	https://medba.ddtmlab.org (accessed on 6 January 2023)	Yu JL et al. (2022) [86]
MolGlueDB	Ocean Univ China	Freely accessible resource for advancing research on MGD	https://www.molgluedb.com (accessed on 21 August 2025)	Wang, X et al. (2025) [87]
ELiAH	Korea Inst Sci & Technol Informat KISTI	Comprehensive repertoire of E3 ligases for ubiquitination-based TPD drug development	https://eliahdb.org/ (accessed on 12 October 2024)	Paik, H et al. (2025) [88]
E3Atlas	Indiana University	Rapidly identify E3 ligases with promising TPD activities against specifically desired targets	https://hanlaboratory.com/E3Atlas/ (accessed on 16 October 2023)	Liu Y et al. (2023) [89]
UbiNet2.0	The Chinese University of Hong Kong	Experimentally validated ESIs and a visualised tool for the construction of ubiquitination regulatory networks	https://academic.oup.com/database/article/doi/10.1093/database/baab010/6162640 (accessed on 8 March 2021)	Li ZY et al. (2021) [90]
UbiHub	University of Toronto	A navigation tool for medicinal chemists, structural and cell biologists exploring ubiquitination pathways	https://ubihub.thesgc.org (accessed on 2 January 2019)	Liu LH et al. (2019) [91]
Enamine’s Linker for Linkerology	Enamine Ltd.	Different linker molecules:17,964 comprehensive linkers; 5233 stock linkers; 12,731 MADE linkers	https://enamine.net/ (accessed on 24 December 2024)	NA
**Models**				
PCG	Cambridge Crystallographic Data Centre	A knowledge-based protocol and tool for modeling the conformational space of chimeric degraders and producing simple rigid-body-assembled TC models.	https://github.com/ccdc-opensource/science-paper-protac-conformer-generator-2025 (accessed on 5 September 2025)	Montisci, F et al. (2025) [92]
MEGA PROTAC	University of Birmingham	MEGA DOCK-based PROTAC mediated ternary complex formation pipeline with sequential filtering and rank aggregation	https://github.com/yauz3/MEGA-PROTAC (accessed on 14 February 2025)	Ugurlu SY et al. (2025) [93]
PROTACable	The University of British Columbia	An Integrative Computational Pipeline of 3-D Modeling and Deep Learning To Automate the De Novo Design of PROTACs	https://github.com/giaguaro/PROTACable/ (accessed on 19 March 2024)	Mslati H et al. (2024) [94]
PROTACability	Université Claude Bernard Lyon	Ability to impact early-stage PROTAC-based drug design campaigns	https://github.com/GilbertoPPereira/PROTACability (accessed on 25 October 2023)	Pereira GP et al. (2023) [95]
PROTAC-Model	Zhejiang University	An integrative computational method by combining the FRODOCK-based protocol and RosettaDock-based refinement to predict PROTAC-mediated ternary complex structures	https://github.com/gaoqiweng/PROTAC-Model (accessed on 28 October 2021)	Wei GQ et al. (2021) [96]
PRosettaC	Weizmann Institute of Science	A combined protocol for the modeling of a ternary complex induced by a given PROTAC.	https://prosettac.weizmann.ac.il/ (accessed on 25 September 2020)	Zaidman D et al. (2020) [97]

MGD: Molecular glue degraders.

**Table 4:** Clinical progress of TPD.

Degrader	Target	E3	Indication	Clinical Phase	Company
**PROTACS**					
Luxdegalutamide (ARV-766) [98]	AR	CRBN	mCRPC	III	Novartis (Basel, Switzerland)
ARV110 [99]	AR	CRBN	mCRPC	II	Arvinas (New Haven, CT, USA)
GT20029	AR	CRBN	AGA	II	Suzhou Kintor Pharmaceutical Inc (Suzhou, Jiangsu Province, China)
CC-94676 [100]	AR	CRBN	mCRPC	I	Celgene (Summit, NJ, USA)
AC176 [101]	AR	CRBN	mCRPC	I	Accutar (Shanghai, China)
HP518 [102,103]	AR	NA	mCRPC	I/II	Hinova (Chengdu, Sichuan Province, China)
Bms-986365 [104]	AR	CRBN	mCRPC	II	Celgene (Summit, NJ, USA)
QLH12016 [105]	AR	NA	mCRPC	I	Qilu Pharmaceutical Co., Ltd. (Jinan, Shandong Province, China)
HRS-5041 [106]	AR	CRBN	mCRPC	I	Jiangsu HengRui Medicine Co., Ltd. (Lianyungang, Jiangsu Province, China)
AH-001 [107]	AR	NA	AGA	I	AnHorn Medicines Co. Ltd. (Shanghai, China)
Vepdegestrant (ARV-471) [108,109]	ER	CRBN	ER- positive, HER2-negative, *ESR1*-mutated advanced or metastatic breast cancer	Approved (FDA, 2026)	Arvinas (New Haven, CT, USA)
HP568 [110]	ER	CRBN	ER+/HER2 Advanced Breast Cancer	I/II	Hinova (Chengdu, Sichuan Province, China)
AC682 [111]	ER	NA	Advanced or Metastatic Breast Cancer	I	Accutar (Shanghai, China)
HRS-1358 [112]	ER	NA	Advanced Breast Cancer	I	Shandong Suncadia Medicine Co., Ltd. (Jinan, Shandong Province, China)
BGB-16673 [113]	BTK	NA	Chronic Lymphocytic Leukemia or Small Lymphocytic Lymphoma	III	BeOne (Shanghai, China)
NX-2127 [114]	BTK	CRBN	B-cell Malignancy	I	Nurix (San Francisco, CA, USA)
NX-5948 [115]	BTK	CRBN	B-cell Malignancy	I	Nurix (San Francisco, CA, USA)
HSK29116 [116]	BTK	NA	B-cell Malignancy	I	Haisco (Harbin, Heilongjiang Province, China)
TQB3019 [117]	BTK	NA	Advanced Malignant Tumors	I	Chia Tai Tianqing (Lianyungang, Jiangsu Province, China)
AC676 [118]	BTK	NA	B-Cell Malignancies	I	Accutar (Shanghai, China)
CFT8634 [119]	BRD9	CRBN	SMARCB1-Perturbed Cancer	I	C4 (Watertown, MA, USA)
FHD-609 [120]	BRD9	NA	SMARCB1-Loss Tumors	I	Foghorn (Cambridge, MA, USA)
MT-4561 [121]	BRD4	NA	Solid Tumors	I/II	Mitsubishi Tanabe Pharma America Inc. (Jersey City, NJ, USA)
LT-002-158 [122]	IRAK4	NA	Hidradenitis Suppurativa	I/II	Leadingtac (Shanghai, China)
KT-474 [123]	IRAK4	CRBN	AD, HS	I	Kymera (Cambridge, MA, USA)
KT-413 [124]	IRAK4	CRBN	B-cell NHL	I	Kymera (Cambridge, MA, USA)
BGB-45035 [125]	IRAK4	NA	Autoimmune Dermatological Diseases	I	BeiGene (Beijing, China; Somerville, MA, USA)
CFT8919 [126]	EGFR	CRBN	NSCLC	I	Betta (Hangzhou, Zhejiang Province, China)
HSK40118 [127]	EGFR	NA	NSCLC	I	Haisco (Harbin, Heilongjiang Province, China)
BG-60366 [128]	EGFR	NA	NSCLC	I	BeiGene (Beijing, China; Somerville, MA, USA)
BMS-986458 [129]	BCL6	NA	Non-Hodgkin Lymphomas	I/II	Bristol-Myers Squibb (New York, NY, USA)
ARV-393 [130]	BCL6	NA	Non-Hodgkin Lymphoma	I	Arvinas ((New Haven, CT, USA))
ASP3082 [131]	KRAS	NA	Solid Tumors	I	Astellas (Tokyo, Japan)
PT0253 [132]	KRAS	NA	Solid Tumors	I	PAQ (Boston, MA, USA)
ASP4396 [133]	KRAS	NA	Solid Tumors	I	Astellas (Tokyo, Japan)
DT2216 [134]	BCL-XL	VHL	Solid Tumors and Fibrolamellar Carcinoma	I/II	Children’s Oncology Group (Monrovia, CA, USA)
KT-333 [135]	STAT3	NA	Refractory Lymphoma, Large Granular Lymphocytic Leukemia, Solid Tumors	I	Kymera (Cambridge, MA, USA)
CFT1946 [136]	BRAF	CRBN	BRAF V600 Mutant Solid Tumors	I	C4 (Watertown, MA, USA)
PRT3789 [137]	SMARCA2	NA	Solid Tumors	II	Prelude (Wilmington, DE, USA)
BMS-986470 [138]	WIZ/ZBTB7A	NA	Sickle Cell Disease	I/II	Bristol-Myers Squibb (New York, NY, USA)
AUTX-703 [139]	KAT2	NA	AML and MDS	I	Auron (Boston, MA, USA)
KT-621 [140]	STAT6	NA	Atopic Dermatitis	II	Kymera (Cambridge, MA, USA)
KT-253 [141]	MDM2	NA	High Grade Myeloid Malignancies, Acute Lymphocytic Leukemia, Lymphoma, Solid Tumors	I	Kymera (Cambridge, MA, USA)
AXT-1003 [142]	EZH2	NA	Non-Hodgkin Lymphomas	I	Axter (Cambridge, MA, USA)
NKT3964 [143]	CDK2	NA	Solid Tumors	I	NiKang (Suzhou, Jiangsu Province, China)
BTX-9341 [144]	CDK4/6	NA	Breast Cancer	I	Biotheryx (Seattle, WA, USA)
**Molecular glue degraders**					
CC-99282 [145]	IKZF1/3	CRBN	Lymphoma	II	M.D. Anderson Cancer Center (Houston, TX, USA)
CC-220 [146,147,148]	IKZF1/3	CRBN	SLE, Skin Sarcoidosis, Lymphomas, Multiple Myeloma	I/II	Celgene (Summit, NJ, USA)
CC-92480 [149]	IKZF1/3	CRBN	RRMM	I/II	Celgene (Summit, NJ, USA)
KPG-818 [150]	IKZF1/3	CRBN	SLE, Hematological Malignancies	I/II	Kangpu (Shanghai, China)
CC-122 [151]	IKZF1/3	CRBN	Advanced Solid Tumors, Non-Hodgkin’s Lymphoma, Multiple Myeloma	I	Celgene (Summit, NJ, USA)
CFT7455 [152]	IKZF1/3	CRBN	Relapsed/Refractory Non-Hodgkin’s Lymphoma or Multiple Myeloma	I	C4 (Watertown, MA, USA)
BTX-1188 [153]	IKZF1/3	CRBN	Advanced Malignancies	I	Biotheryx (Seattle, WA, USA)
KPG-121 [154]	IKZF1/3	CRBN	CRPC	I	Kangpu (Shanghai, China)
DKY709 [155]	IKZF1/3	CRBN	Solid Tumors	I	Novartis (Basel, Switzerland)
TQB3820 [156]	IKZF1/3	CRBN	Hematological Malignancies	I	Chia Tai Tianqing (Lianyungang, Jiangsu Province, China)
MRT-2359 [157]	GSPT1	CRBN	Lung Cancer and Diffuse B-Cell Lymphoma	I/II	Monte Rosa (Basel, Switzerland)
CC-90009 [158]	GSPT1	CRBN	Myeloid Leukemia	I	Celgene (Summit, NJ, USA)
E7820 [159]	RBM39	DCAF15	Solid Tumors	II	Eisai (Tokyo, Japan)
ICP-490 [160]	IKZF1/3	CRL4	Multiple Myeloma	I/II	Beijing InnoCare (Beijing, China)
**Autophagy-Targeting Chimeras**					
ATG101 [161]	PD-L1	NA	Solid Tumors and Mature B-cell Non-Hodgkin Lymphomas	I	Antengene (Shanghai, China)

mCRPC: Metastatic Castration Resistant Prostate Cancer. AGA: Androgenetic Alopecia. RRMM: Relapsed and Refractory Multiple Myeloma. SLE: Systemic Lupus Erythematosus. AD: Atopic Dermatitis. HS: Hidradenitis Suppurativa. NSCLC: Non-small Cell Lung Cancer. AML: Acute Myeloid Leukemia. MDS: Myelodysplastic Syndromes.

## Data Availability

The original contributions presented in this study are included in the article/Supplementary Materials. Further inquiries can be directed to the corresponding authors.

## Supplementary Materials

The following supporting information can be downloaded at: https://www.mdpi.com/article/10.3390/pharmaceutics18070887/s1, Figure S1: Heatmap of institutions; Figure S2: Timezone view of institutions; Figure S3: Most locally cited sources of journals; Figure S4: Timezone view of cited reference; Figure S5: Timeline view of keywords clusters; Figure S6: Temporal evolution of 2001–2005 research topics in TPD; Figure S7: Temporal evolution of 2006–2010 research topics in TPD; Figure S8: Temporal evolution of 2011–2015 research topics in TPD; Figure S9: emporal evolution of 2016–2020 research topics in TPD; Figure S10: Temporal evolution of 2021–2025 research topics in TPD; Table S1: Search strategy; Table S2: Top 9 institutions by Sigma value (research novelty); Table S3: Top ten productive corresponding authors; Table S4: Top ten productive journals; Table S5: Summary of the largest 19 clusters of cited.
