# Supplementary material for: Mapping Scientific Landscapes and Therapeutic Innovations of Targeted Protein Degradation: A Scientometric Review
Source: Pharmaceutics. 2026 Jul 20;18(7):887. doi: 10.3390/pharmaceutics18070887 (PMC13414865; doi:10.3390/pharmaceutics18070887)
Supplement: Supplementary file 1 [file pharmaceutics-18-00887-s001.zip › pharmaceutics-4344711-supplementary.pdf]

Supplementary Materials: Mapping Scientific Landscapes and Therapeutic Innovations of Targeted Protein Degradation: A Scientometric Review

Chong Li, Xiangxiu Wang, Anqi He, Tianjie Bao, Weihua Zhuang, Chengqi He and Yonghong Yang

Table S1. Search strategy.

Step	Search Fields & Topic Terms
#1	**PROTACs & Bifunctional Degraders:** TS = ("proteolysis targeting chimera*" OR "proteolysis-targeting chimera*" OR "PROTAC*" OR "bifunctional degrader*" OR "heterobifunctional degrader*" OR "SNIPER*" OR "specific and non-genetic inhibitor* of apoptosis protein* dependent nocturnal O2 degradation protein*")
#2	**Molecular Glues:** TS = ("molecular glue*" OR "degradation inducer*" OR "immunomodulatory drug*" OR "IMiD*" OR "thalidomide analog*" OR "lenalidomide" OR "pomalidomide")
#3	**Endosome/Lysosome & Autophagy-Targeting Chimeras:** TS = ("LYTAC*" OR "lysosome* targeting chimera*" OR "AUTAC*" OR "autophagy* targeting chimera*" OR "ATTEC*" OR "autophagy tethering compound*" OR "AUTOTAC*" OR "ribonuclease targeting chimera*" OR "RIBOTAC*")
#4	**General Targeted Degradation Terms:** TS = (("targeted protein degradation" OR "selective protein degradation" OR "induced protein degradation") AND ("ubiquitin*" OR "proteasome*" OR "lysosome*" OR "autophagy"))
#5	**Combined TPD Dataset:** **#1 OR #2 OR #3 OR #4**
#6	**Refined by Document Type & Language:** #5 AND Document Types: (Article OR

	Review) AND Languages: (English)

Table S2. Top 9 institutions by Sigma value (research novelty).

Institution	Sigma	Centrality	Bursts
Yale University	20.23	0.1	30.21
Howard Hughes Medical Institute	2.09	0.19	4.71
St Jude Children's Research Hospital	1.51	0.12	7.53
Zhengzhou University	1.41	0.09	7.26
University of California System	1.38	0.09	4.71
University of Tokyo	1.35	0.04	7.05
Kyoto Prefectural University of Medicine	1.34	0.03	6.19
UTMD Anderson Cancer Center	1.34	0.05	6.09
University of Dundee	1.32	0.04	7.26

Table S3. Top ten productive corresponding authors.

Author	H_index	TC	NP	PY_start	Institution
Crews, Craig M	51	19596	70	2001	Yale University
Alessio Ciulli	29	5399	45	2015	University of Dundee
Eric Fischer	25	3412	40	2016	Harvard Medical School
Liu, Jing	25	2443	62	2018	Xi'an Jiaotong University
Jian Jin	24	2592	58	2018	Icahn School of Medicine at Mount Sinai
Wang Jing	23	5000	61	2015	Arvinas Operat Inc
Liu Yang	22	2009	70	2020	Shenyang Pharmaceutical University
Katherine Aleisha Donovan	21	2292	34	2017	Icahn School of Medicine at Mount Sinai
Wang Shaomeng	21	2674	41	2016	University of Michigan
Zhang Xuan	21	2185	37	2019	University of Chinese Academy of Sciences

TC = total citations; NP = total number of papers published; PY = publication year.

Table S4. Top ten productive journals.

Journals	WoS Categories	Category Rank	IF (2024)	h_index	TC	NP	PY_start
JOURNAL OF MEDICINAL CHEMISTRY	CHEMISTRY, MEDICINAL	7/72	6.8	55	10693	313	2016
EUROPEAN JOURNAL OF MEDICINAL CHEMISTRY	CHEMISTRY, MEDICINAL	10/72	5.9	40	4997	195	2018
JOURNAL OF THE AMERICAN CHEMICAL SOCIETY	CHEMISTRY, MULTIDISCIPLINARY	17/239	15.7	30	3996	83	2004
ACS CHEMICAL BIOLOGY	BIOCHEMISTRY & MOLECULAR BIOLOGY	120/320	3.8	29	4200	58	2015
CELL CHEMICAL BIOLOGY	BIOCHEMISTRY & MOLECULAR BIOLOGY	40/320	7.2	27	4179	53	2016
NATURE COMMUNICATIONS	MULTIDISCIPLINARY SCIENCES	10/136	15.7	26	3420	75	2017
ANGEWANDTE CHEMIE-INTERNATIONAL EDITION	CHEMISTRY, MULTIDISCIPLINARY	15/239	17	23	2958	58	2016
NATURE CHEMICAL BIOLOGY	BIOCHEMISTRY & MOLECULAR BIOLOGY	12/320	13.7	21	5731	32	2015
CHEMICAL COMMUNICATIONS	CHEMISTRY, MULTIDISCIPLINARY	84/239	4.2	20	1516	32	2017
ACS MEDICINAL CHEMISTRY LETTERS	CHEMISTRY, MEDICINAL	25/72	4	19	1286	51	2018

TC = total citations; NP = total number of papers published; PY = publication year.

Table S5. Summary of the largest 19 clusters cited.

Cluster ID	Size	Silhouette	Label (LLR)	Average Year
0	100	0.801	potent selective	2019
1	92	0.908	molecular glue degrader	2019
2	85	0.874	e3 ligases	2020
5	78	0.916	protac-mediated ternary complexe	2017
3	76	0.895	silico design	2021
4	58	0.836	boc3arg-linked ligand	2014
7	53	0.969	membrane protein	2021
8	49	0.932	acid-based targeted degradation	2021
10	46	0.871	protein degradation inducer	2015
11	46	0.902	dissolution performance	2022
12	46	0.89	histone deacetylase	2020
13	39	0.885	streamlined development	2020
15	36	0.955	molecular glue degrader	2021
16	32	0.952	targeted protein degradation	2020
17	24	0.955	medicinal chemists perspective	2018
6	21	1	degradation	2000
9	20	0.994	useful chemical	2005
14	19	0.968	protac-induced bet protein degradation	2014
22	5	0.997	rapid depletion	2009

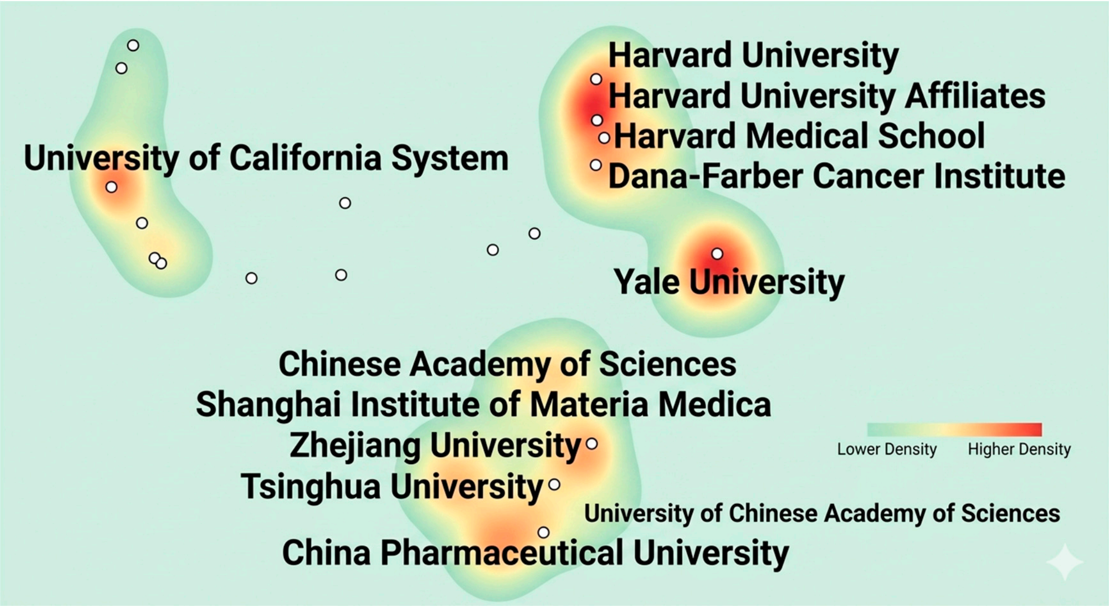

Figure S1. Heatmap of institutions.

.

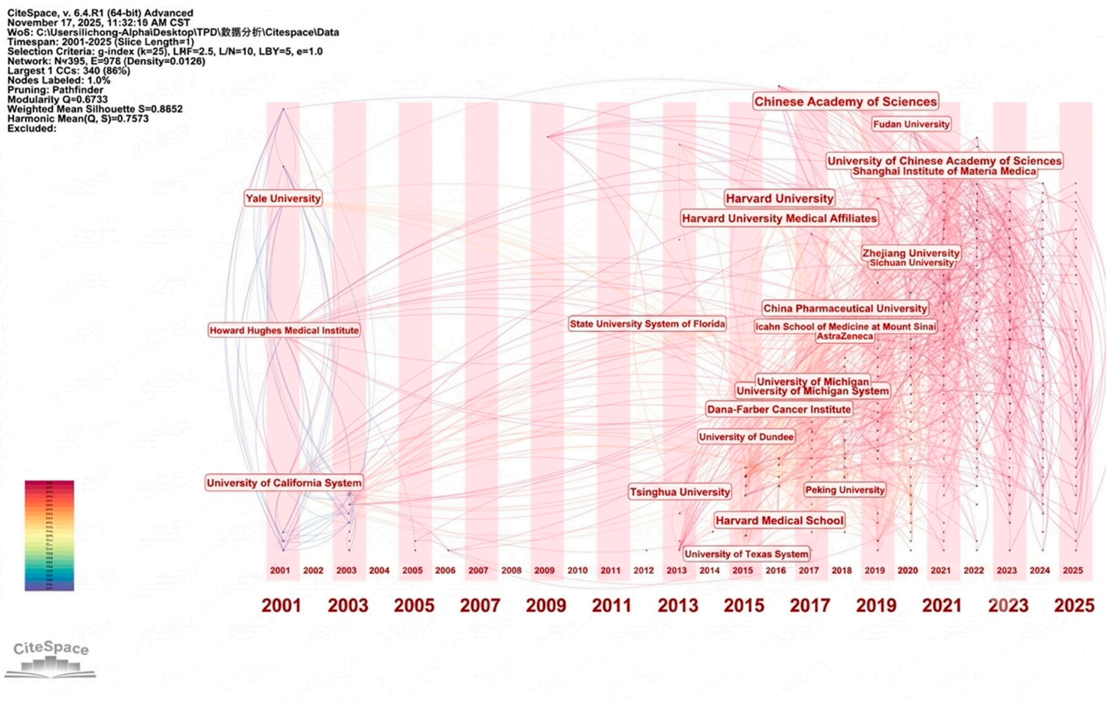

Figure S2. Timezone view of institutions.

.

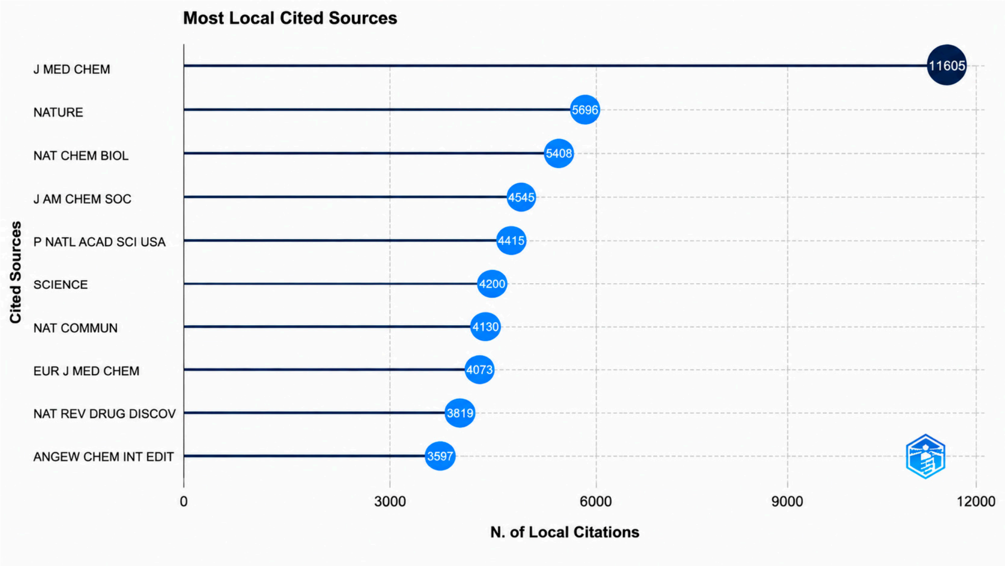

Figure S3. Most locally cited sources of journals.

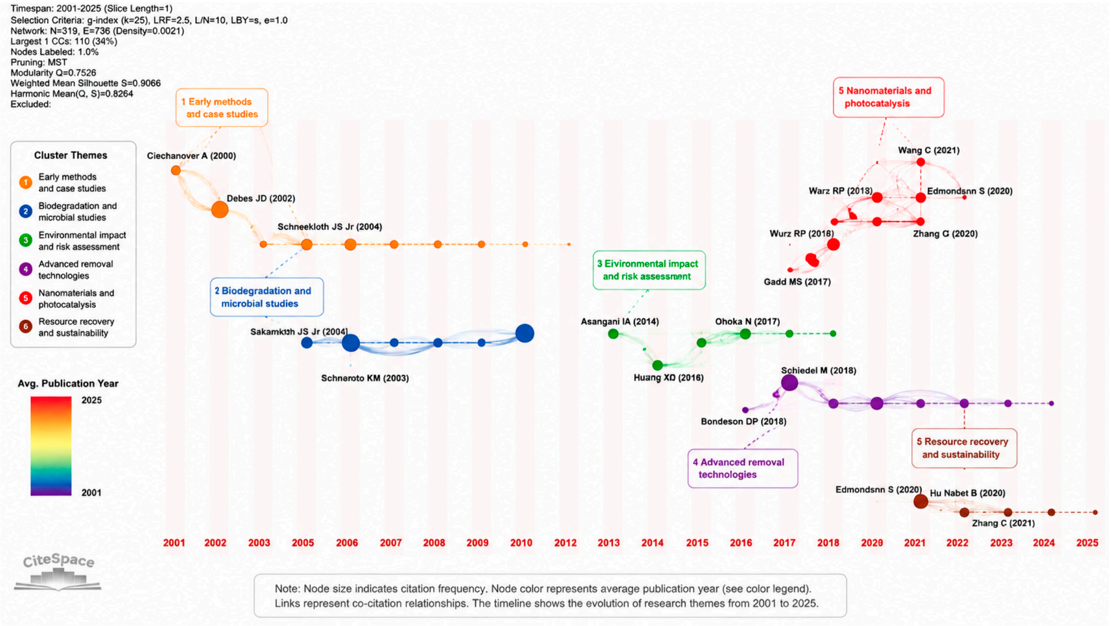

Figure S4. Timezone view of cited reference.

.

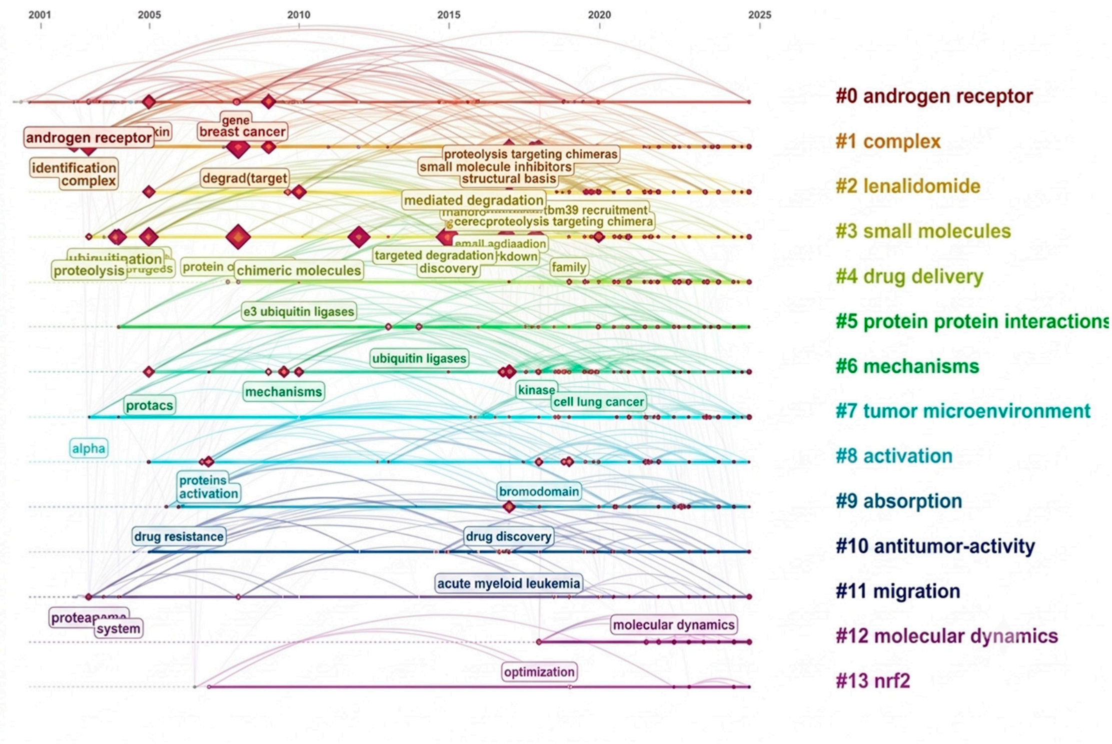

Figure S5. Timeline view of keywords clusters.

.

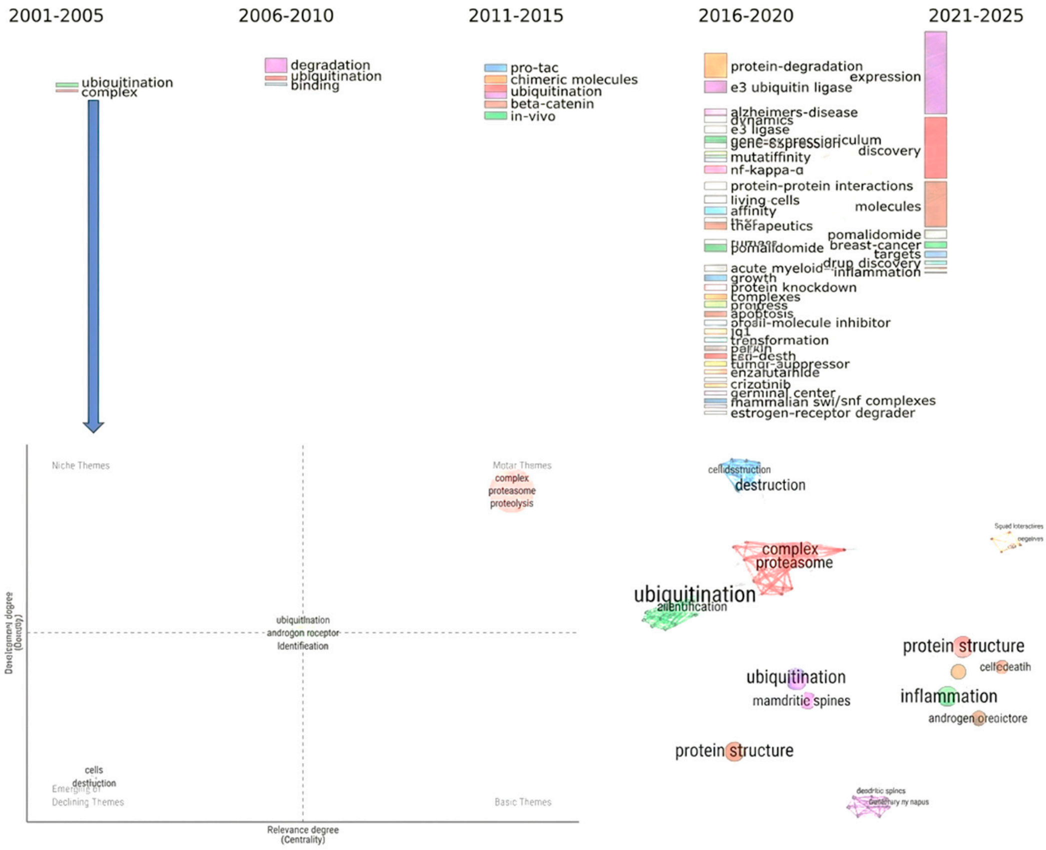

Figure S6. Temporal evolution of 2001-2005 research topics in TPD.

.

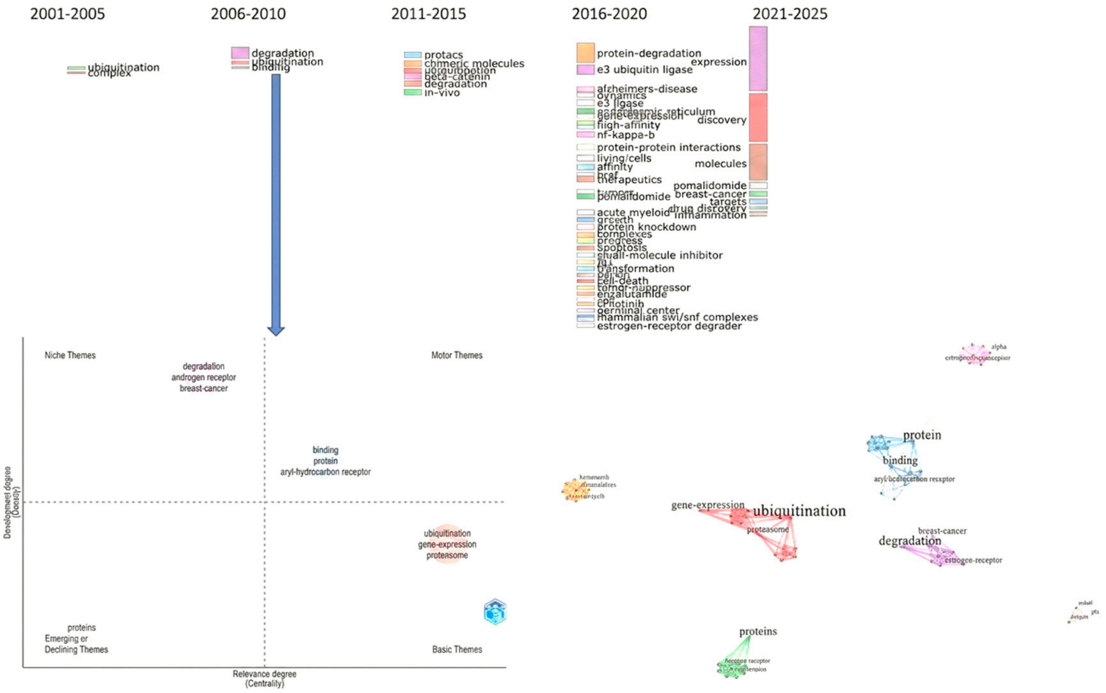

Figure S7. Temporal evolution of 2006-2010 research topics in TPD.

.

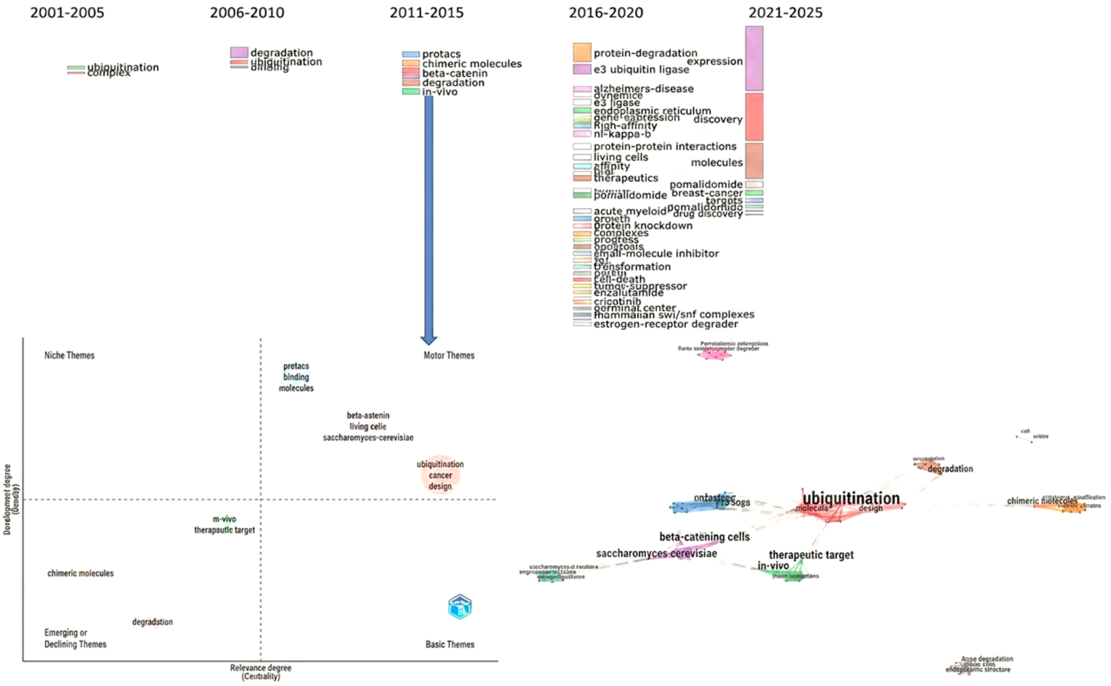

Figure S8. Temporal evolution of 2011-2015 research topics in TPD.

.

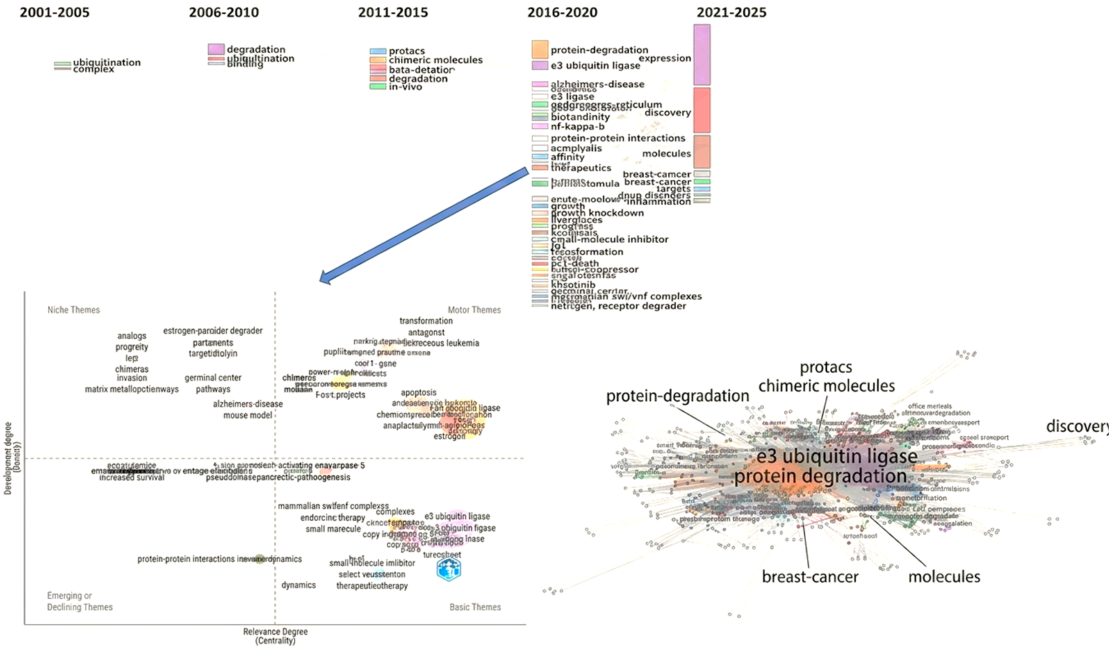

Figure S9. Temporal evolution of 2016-2020 research topics in TPD.

.

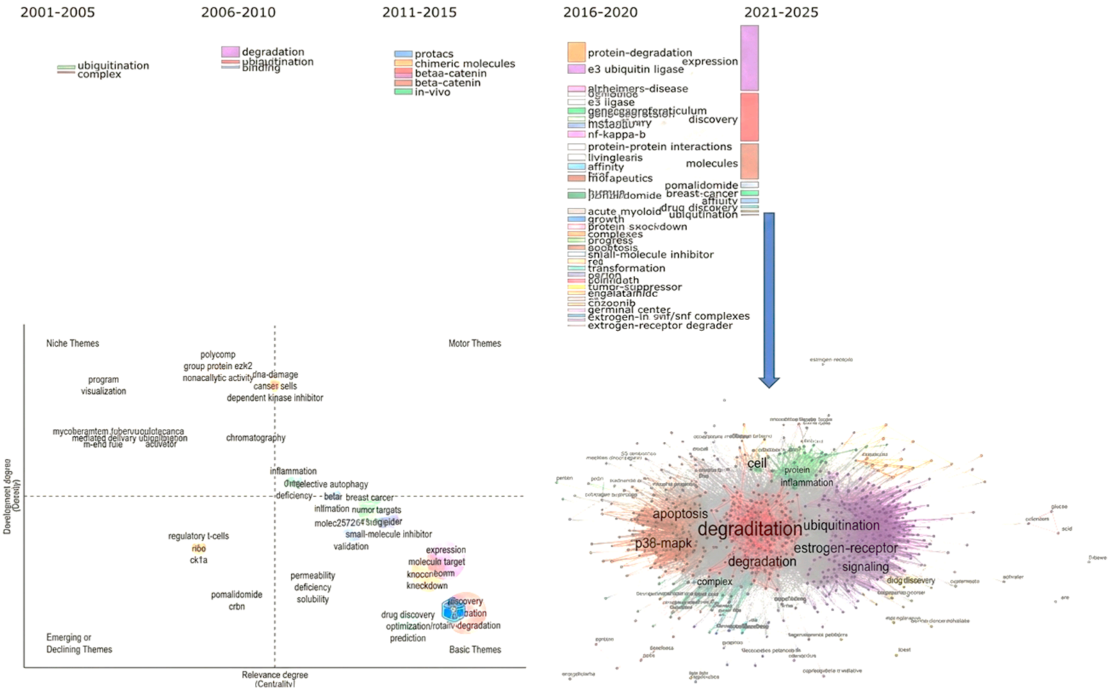

Figure S10. Temporal evolution of 2021-2025 research topics in TPD.
